# Supplementary material for: Reconstructing promoter activity from Lux bioluminescent reporters
Source: PLoS Comput Biol. 2017 Sep 18;13(9):e1005731. doi: 10.1371/journal.pcbi.1005731 (PMC5619816; doi:10.1371/journal.pcbi.1005731)
Supplement: S1 Text — (PDF) [file pcbi.1005731.s001.pdf]

S1 Text: Reconstructing promoter activity from Lux bioluminescent reporters

August 31, 2017

Mudassar Iqbal, Neil Doherty, Anna M.L. Page, Saara N.A. Qazi, Ishan Ajmera, Peter A. Lund, Theodore Kypraios, David J. Scott, Philip J. Hill & Dov J. Stekel

S1 Text

This document contains detailed methods including reactions mechanisms and related velocity derivations, additional figures for model data fits, as well as diagnostic figures for MCMC method for parameter estimation for all three reactions (Fre, LuxAB, and LuxEC).

1 Methods

1.1 An improved biochemical model for the Lux reactions

For all the reactions, we present the detailed mechanisms and derive the velocity equations using King and Altman's schematic method (King and Altman 1956).

Fre Reaction: Flavin recycling

The Fre-catalysed FMN/NADPH reaction, given by E.C.1.5.1.29, is:

$$\mathrm{FMN} + \mathrm{NADPH} + \mathrm{H}^{+} \xrightarrow{\mathrm{Fre}} \mathrm{FMNH_2} + \mathrm{NADP}^{+} \tag{1}$$

The mechanism used for this reaction is given by:

$$\left.\begin{aligned} \mathrm{Fre} &\underset{k_{-1}}{\overset{k_1[FMN]}{\rightleftharpoons}} \mathrm{Fre\cdot FMN} \\ \mathrm{Fre\cdot FMN} &\xrightarrow{k_2[NADPH]} \mathrm{Fre\cdot FMN\cdot NADPH} \\ \mathrm{Fre\cdot FMN\cdot NADPH} &\xrightarrow{k_3} Fre + ... \end{aligned}\right\} \tag{2}$$

This mechanism is used to derive a velocity equation for the reaction. In this and other velocity equations, two parameter conventions are used: $k_1^r = \frac{k_{-1}}{k_1}$ for reversibility constants; and $k_b^a = \frac{k_a}{k_b}$ for other reaction parameters.

$$v_{Fre} = \frac{V_{max}[FMN][NADPH]}{k_1^r k_2^3 + k_2^3[FMN] + k_1^3[NADPH] + [FMN][NADPH]} \quad (3)$$

LuxAB Reaction: Main light pathway

The two lux genes luxA and luxB encode for luciferase, which, in the presence of aldehyde, catalyses the reaction involving oxidation of $FMNH_2$ to oxidised flavin (FMN) and the emission of light. The reaction, given by E.C.1.14.13.3, is:

$$\mathrm{FMNH_2 + O_2 + RCHO \xrightarrow{LuxAB} FMN + H_2O + RCOOH + Light(490nm)} \quad (4)$$

The mechanism used for the derivation of velocity equation is:

$$\left.\begin{array}{r} \mathrm{LuxAB} \underset{k_{-1}}{\overset{k_1[FMNH_2]}{\rightleftharpoons}} \mathrm{LuxAB \cdot FMNH_2} \\ \mathrm{LuxAB \cdot FMNH_2} \xrightarrow{k_2[O_2]} \mathrm{LuxAB \cdot FMNH_2 \cdot O_2} \\ \mathrm{LuxAB \cdot FMNH_2 \cdot O_2} \xrightarrow{k_3[RCHO]} LuxAB.FMNH_2.O_2 - RCHO \\ \mathrm{LuxAB \cdot FMNH_2 \cdot O_2{-}RCHO} \xrightarrow{k_4} \mathrm{LuxAB} + ... \end{array}\right\} \quad (5)$$

The reaction velocity is:

$$v_{LuxAB} = \frac{V_{max}[FMNH_2][O_2][RCHO]}{\left\{\begin{array}{l} k_1^r k_2^4[RCHO] + k_1^4[O_2][RCHO] + k_2^4[FMNH_2][RCHO] \\ + k_3^4[FMNH_2][O_2] + [FMNH_2][O_2][RCHO] \end{array}\right\}} \quad (6)$$

The LuxAB model given in Equation 6 has been refined in the light of the experimental findings suggesting product inhibition by FMN. Because the exact mechanism is as yet unknown, we have included a generic product inhibition term to Equation 6 to obtain the refined velocity equation:

$$v_{LuxAB} = \frac{V_{max}[FMNH_2][O_2][RCHO]}{\left\{\begin{array}{l} k_1^r k_2^4[RCHO] + k_1^4[O_2][RCHO] \\ + k_2^4[FMNH_2][RCHO] \\ + k_3^4[FMNH_2][O_2] + [FMNH_2][O_2][RCHO] \end{array}\right\} * (K_F + FMN)} \quad (7)$$

where K_F is the new parameter associated with product inhibition.

LuxEC Reaction: Aldehyde recycling

The recycling fatty acid back to aldehyde is modelled as a combined reaction catalysed by the LuxEC complex. The reaction combines E.C. 6.2.1.19 and E.C. 1.2.1.50. and is:

$$\mathrm{RCOOH + ATP + NADPH \xrightarrow{LuxEC} RCHO + AMP + NAD^+} \quad (8)$$

The mechanism is:

$$\left.\begin{aligned} \text{LuxEC} \underset{k_{-1}}{\overset{k_1[RCOOH]}{\rightleftharpoons}} \text{RCOOH} \cdot \text{LuxEC} \\ \text{RCOOH} \cdot \text{LuxEC} \xrightarrow{k_2[ATP]} \text{RCOOH} \cdot \text{LuxEC} \cdot \text{ATP} \\ \text{RCOOH} \cdot \text{LuxEC} \cdot \text{ATP} \xrightarrow{k_3} RCO - LuxEC + ... \\ \text{RCO}-\text{LuxEC} \underset{k_{-4}}{\overset{k_4}{\rightleftharpoons}} \text{LuxEC}-\text{RCO} \\ \text{LuxEC}-\text{RCO} \xrightarrow{k_5[NADPH]} LuxEC - RCO - NADPH \\ \text{LuxEC}-\text{RCO}-\text{NADPH} \xrightarrow{k_6} \text{LuxEC} + ... \end{aligned}\right\} \tag{9}$$

The velocity equation associated with this mechanism is:

$$v_{LuxEC} = \frac{V_{max}[RCOOH][ATP][NADPH]}{\left\{\begin{aligned} & k_1^r k_2^6[NADPH] + k_1^6[ATP][NADPH] \\ & + k_2^6[RCOOH][NADPH] + 2k_3^6[RCOOH][ATP][NADPH] \\ & + 2k_4^6[RCOOH][ATP] + [RCOOH][ATP][NADPH] \end{aligned}\right\}} \tag{10}$$

1.2 Sub-models used for model parameter inference

Fre model parameter inference

$$\left.\begin{aligned} \frac{\mathrm{d[NADPH]}}{\mathrm{d\,t}} = -V_{Fre} - \gamma_N[NADPH] \\ \frac{\mathrm{d[FMN]}}{\mathrm{d\,t}} = -V_{Fre} \end{aligned}\right\} \tag{11}$$

In equation 11, γ_N is the *in vitro* degradation parameter for NADPH and V_{Fre} is the rate law for this reaction, given in equation 3. This model is used to simulate the time course behaviour of NADPH and FMN for given values of the parameters which we will infer using the data given in Figure A(a). Parameters to be inferred are given in table 1, where the mean values and standard deviations from the posterior distributions of the MCMC are also shown. The posterior distributions from this experiment are then used as prior distributions to refit these parameters to the LuxAB data set (Figure A(b)), in which the Fre reaction is also involved. Thus the final posterior distributions take into account data from both experiments; these are shown in the second row of Table 1. The full posterior distributions of these parameters with respect to the priors, as well as other MCMC diagnostic information, are provided in the Supplementary figures in relevant sections below.

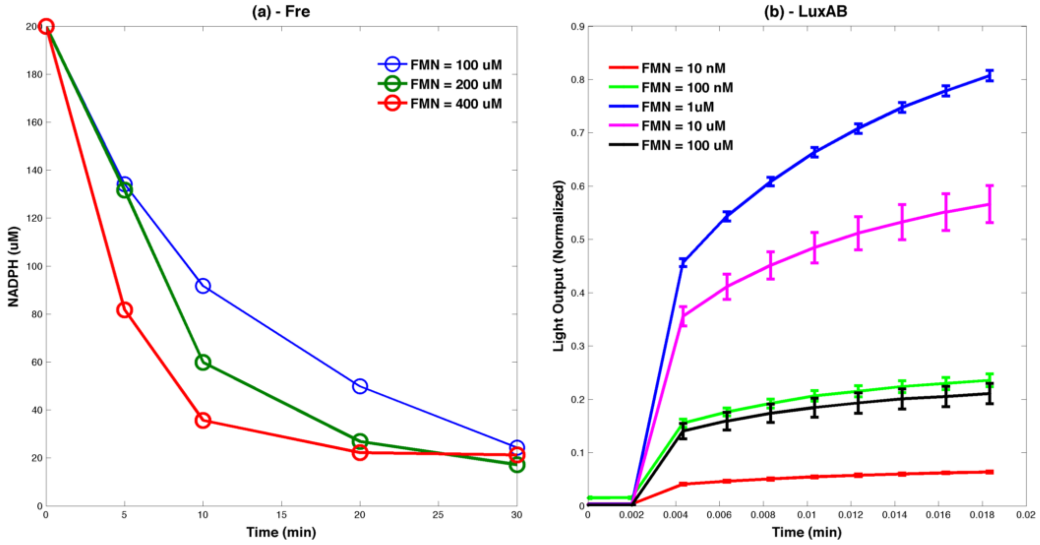

Figure A: Experimental data used for estimation of parameters of Fre and LuxAB models

LuxAB model parameter inference

Parameter inference for the LuxAB reaction was carried out using an MCMC scheme, with the posterior estimates for the Fre parameters as prior estimates for these same parameters in this model. Table 1 details inferred values of LuxAB reaction related parameters. Figure F shows that model fits to data are very good; importantly, the model faithfully reproduces the apparent product inhibition observed in the data. MCMC diagnostics are shown in Supplementary Figures corresponding to LuxAB reaction. The equations used for this inference are given by:

$$\left.\begin{aligned}\frac{\mathrm{d[NADPH]}}{\mathrm{d\,t}} &= -V_{Fre} - \gamma_N[NADPH]\\ \frac{\mathrm{d[FMNH_2]}}{\mathrm{d\,t}} &= V_{Fre} - V_{LuxAB} - \gamma_{F2}[FMNH_2]\\ \frac{\mathrm{d[RCHO]}}{\mathrm{d\,t}} &= V_{LuxAB} - gamma_R[RCHO]\\ \frac{\mathrm{d[FMN]}}{\mathrm{d\,t}} &= -V_{Fre} - \gamma_F[FMN]\end{aligned}\right\} \tag{12}$$

LuxEC model parameter inference

$$\left.\begin{aligned}\frac{\mathrm{d[AMP]}}{\mathrm{d\,t}} &= LuxEC * V_{LuxEC}\\ \frac{\mathrm{d[RCOOH]}}{\mathrm{d\,t}} &= -LuxEC * V_{LuxEC}\end{aligned}\right\} \tag{13}$$

where the reaction velocity is as is shown in equation 10, and which contains the parameters to be inferred.

Table A: Lux Model Parameters, including kinetic constants and fixed concentrations

**Fre**	$V_{max} * Fre$:61.1 (32.9)	K_1^r : 1.0	K_1^3:1.8 (1.4)	K_2^3:45.2 (38.2)	γ_N:0.062 (0.052)	
**LuxAB**	$V_{max} * LuxAB$: 64.97 (45.5)	K_1^r:0.28 (0.23)	K_1^4:0.18 (0.14)	K_2^4:84.5 (60.2)	K_3^4:69.7 (52.1)	K_F:17.1 (7.5)
	γ_N:0.062 (0.052)	γ_{F2}:0.066 (0.056)	γ_R:0.063 (0.051)	γ_F: 0.615 (0.055)		
**LuxEC**	V_max:198.93 (106.1)	K_1^r:0.04 (0.04)	K_1^6:90.9 (77.6)	K_2^6:95.3 (92.6)	K_3^6:24.35 (21.7)	K_3^6:76.5 (62.3)
Others	$Flavin(F)$: 88 uM	$Acid(R)$: 231	O_2:214 uM	$NADPH$: 560 uM	ATP: 1310 uM	$\gamma_L(h^{-1})$: 0.378 (0.04)

2 Supplementrary figures for biochemical reactions parameter inference

2.1 Fre Reaction

Figures B, C, D, E show the detailed model fit for Fre data, and other disgnostic plots like model likeihoods over the course of simulation, traces of the parameters of the Fre model, and prio/posterior plot of individual parameters, respectively.

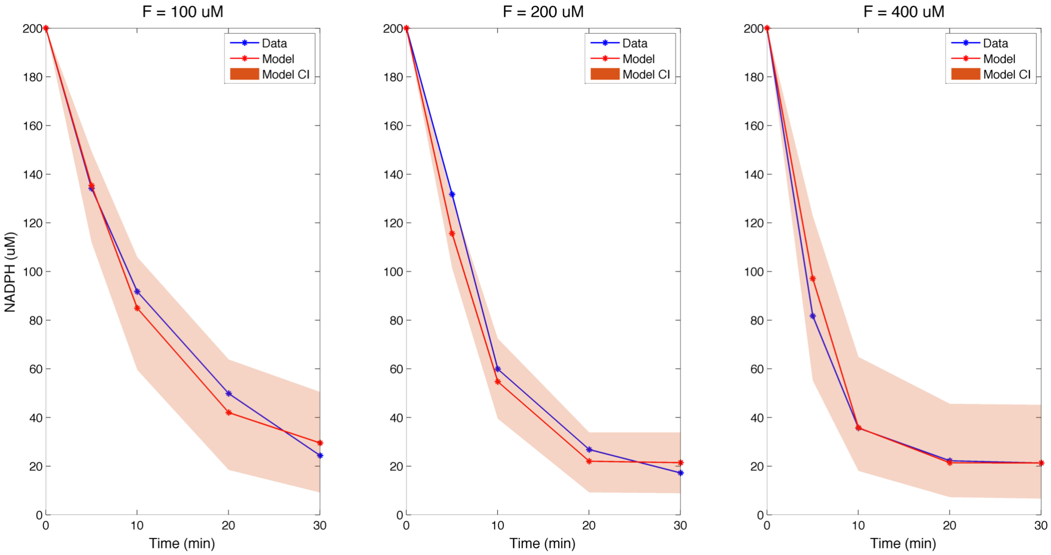

Figure B: Fre reaction: Model fits to experimental data

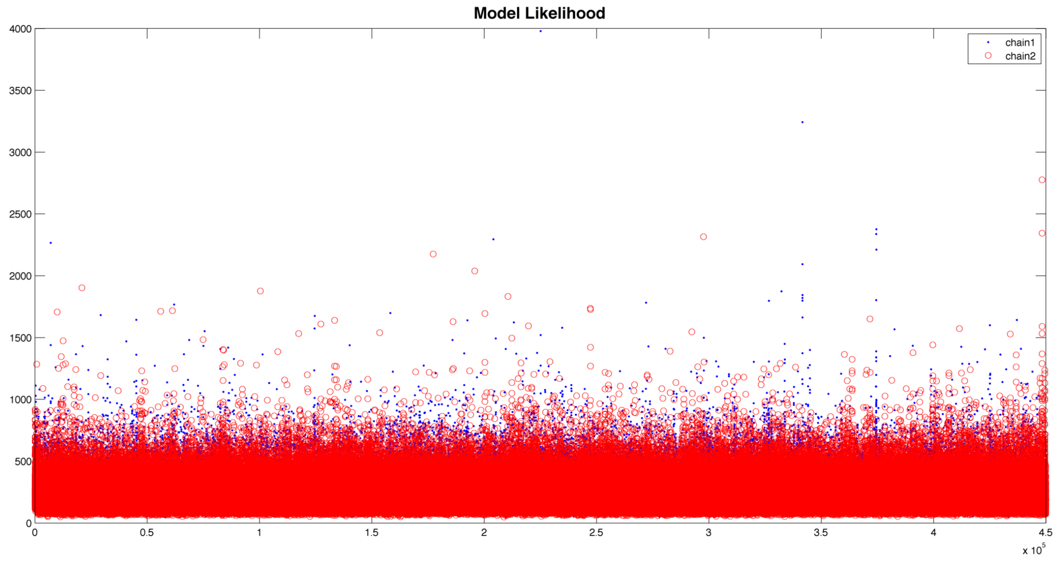

Figure C: Fre reaction: Model likelihood over the course of simulation for two independant MCMC chains

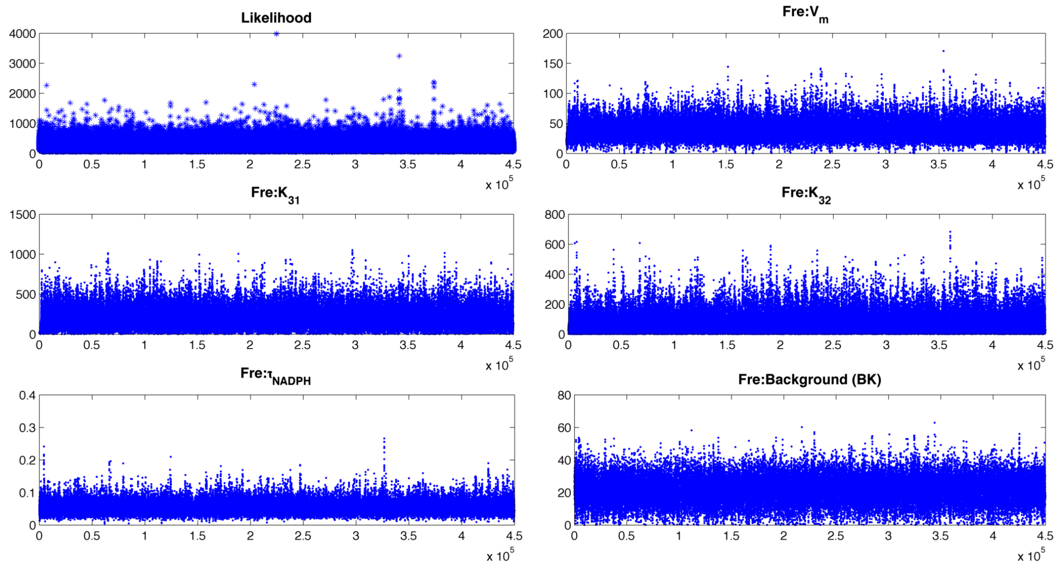

Figure D: Fre reaction: traces (values) for individual parameters of the Fre model during MCMC simulation

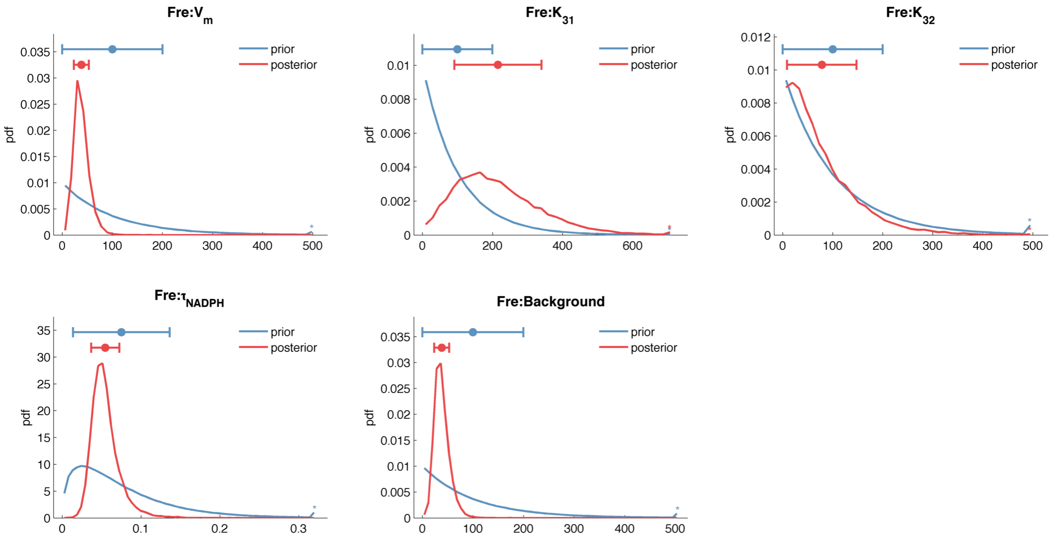

Figure E: Fre reaction: Prior and Posterior histograms for parameters

2.2 LuxAB Reaction

Figure F show the model fit for light output data for different concentration of FMN, while in Figures G, H, I we show additional plots related to MCMC inference for parameters in LuxAB reaction model.

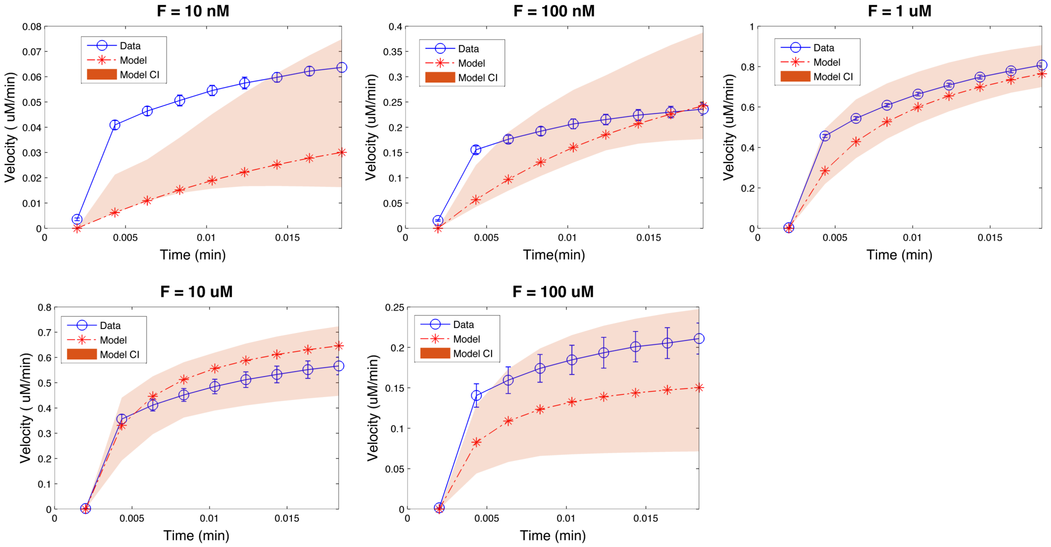

Figure F: LuxAB reaction: Model fits to experimental data

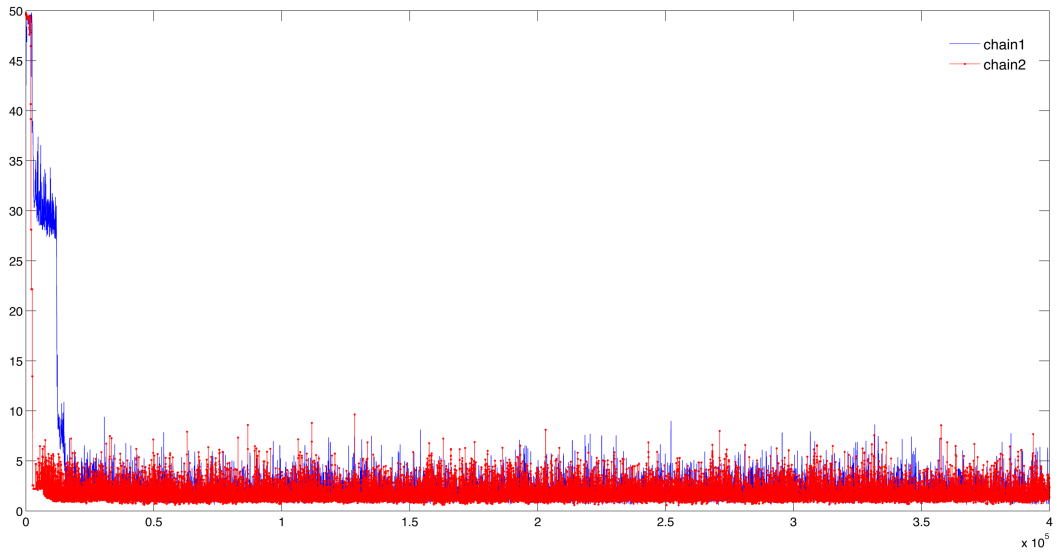

Figure G: LuxAB reaction: Model likelihood over the course of simulation for two independant MCMC chains

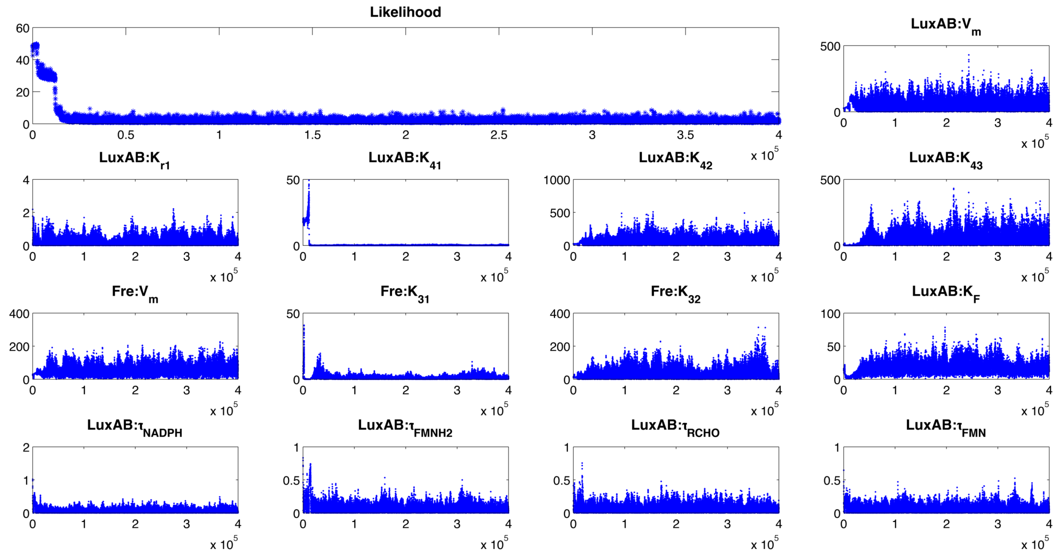

Figure H: LuxAB reaction: traces (values) for individual parameters during MCMC simulation

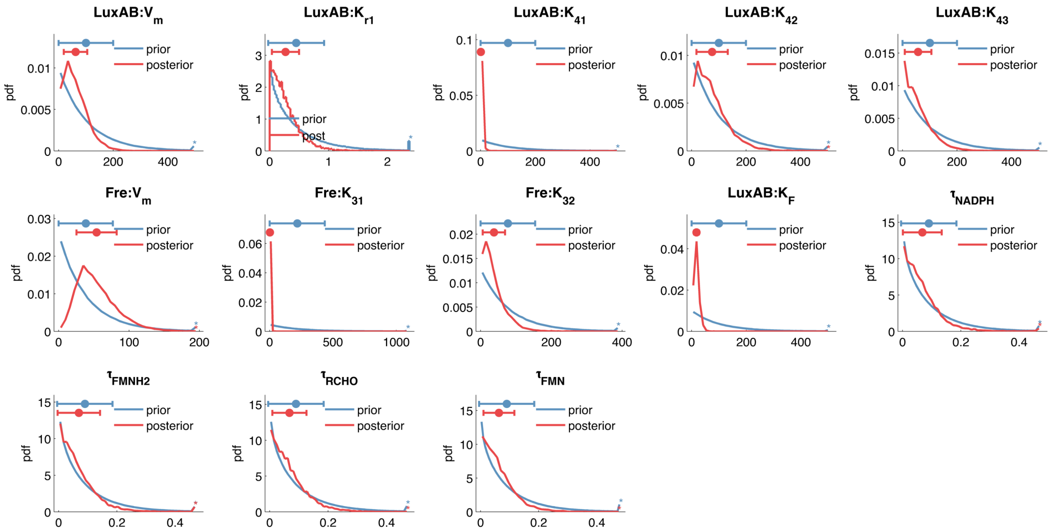

Figure I: LuxAB reaction: Prior and Posterior histograms for parameters

2.3 LuxEC Reaction

Figure J show the model fit for experimental data from literature (as described in main text), while in Figures K,L, M show MCMC output.

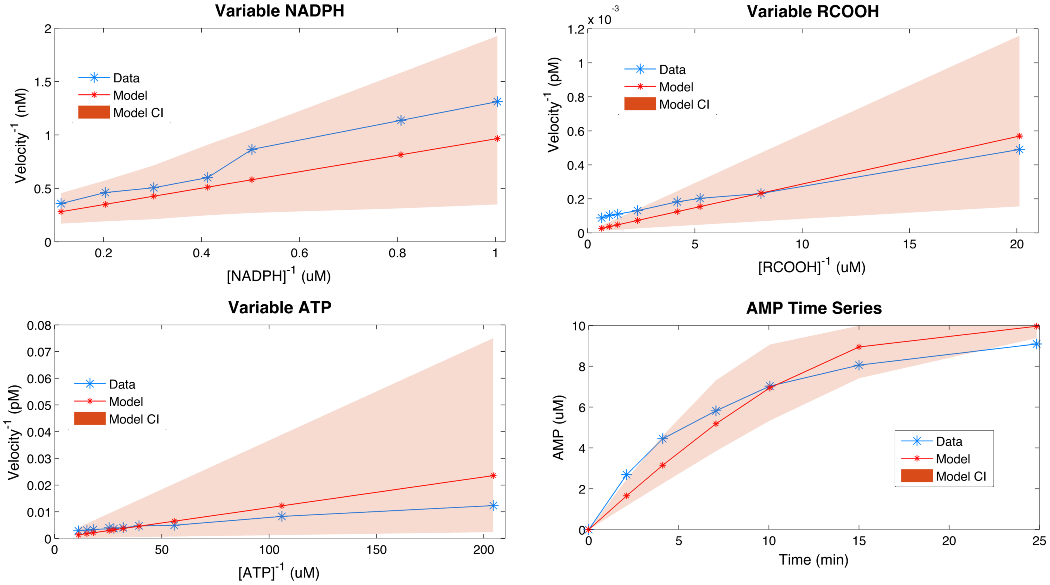

Figure J: LuxEC reaction: Model fits to experimental data

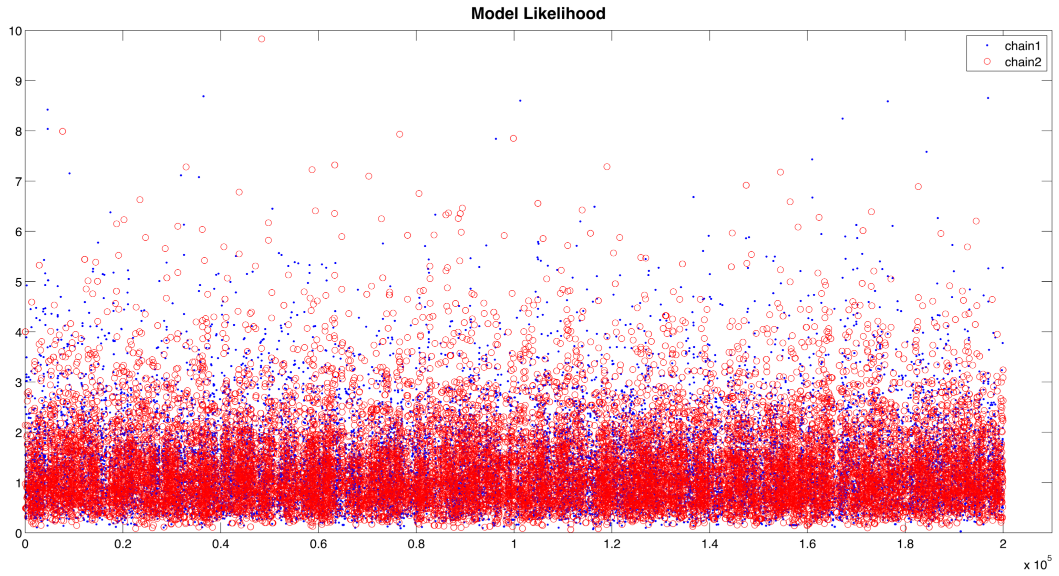

Figure K: LuxEC reaction: Model likelihood for two independant MCMC chains

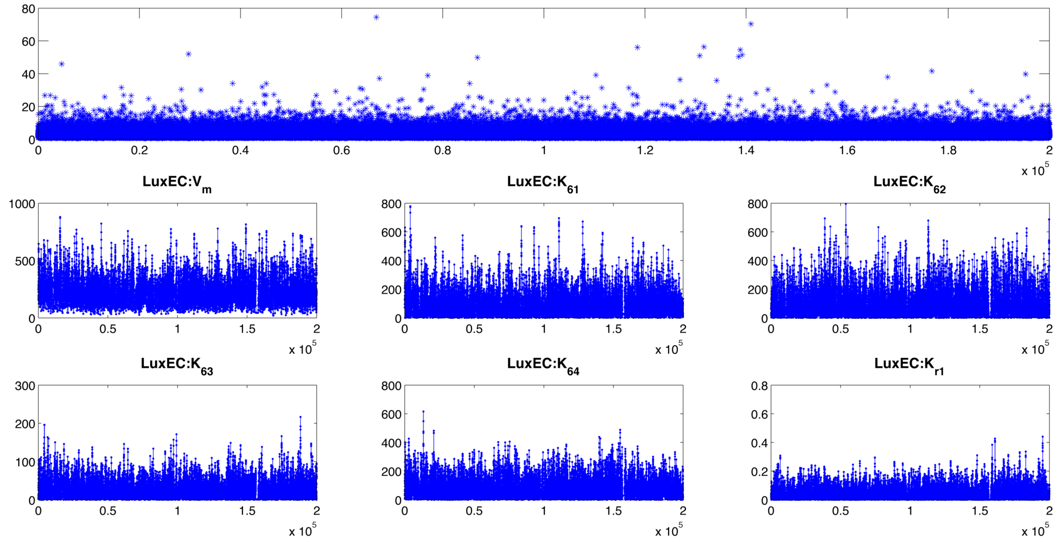

Figure L: LuxEC reaction: traces (values) for individual parameters during MCMC simulation

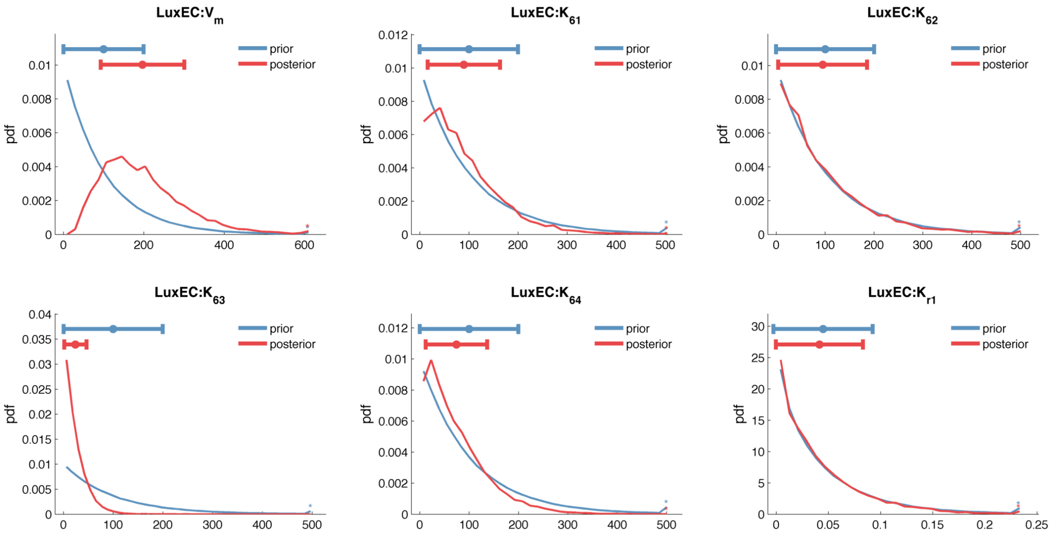

Figure M: LuxEC reaction: Prior and Posterior histograms for parameters

2.4 Convergence Analysis

We have carried out a convergence analysis for kinetic parameters of individual reactions, as well as of height parameters in case of promoter activity inference. Two parallel chains were run for 500K iterations for both kinetic reactions (LuxAB and LuxEC, where LuxAB also includes inference Fre reaction related parameters, as described in the manuscript), and last 100k iterations were used to make posterior estimates, as reported in the manuscript. For promoter activity inference, chains were run for 1 million iterations in total and were sub-sampled (thinned) every 10th iteration, hence we had saved 100K samples for every chains, where last 50K samples were used to make posterior estimates. Given the stored samples, we have calculated convergence diagnostics $\hat{R}$ (Gelman 1992) for all parameters at 15 equally spaced intervals along the chains length (Supplementary Figure N). The chains have converged in the intervals used for estimation of posterior means (given the Gelman's rule of thumb on convergence, i.e., $\hat{R} \leq 1.1$).

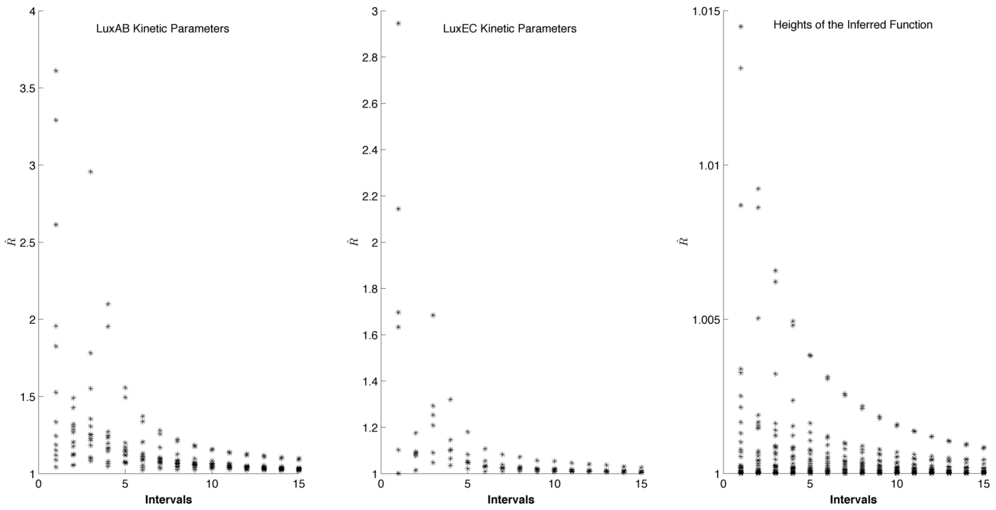

Figure N: $\hat{R}$ Convergence Diagnostics

2.5 TurnOver Rates for Lux Proteins

In Supplementary Figure O, we show the log-linear fit to individual data sets, used for estimation of Lux protein turn over rates, as described in main text.

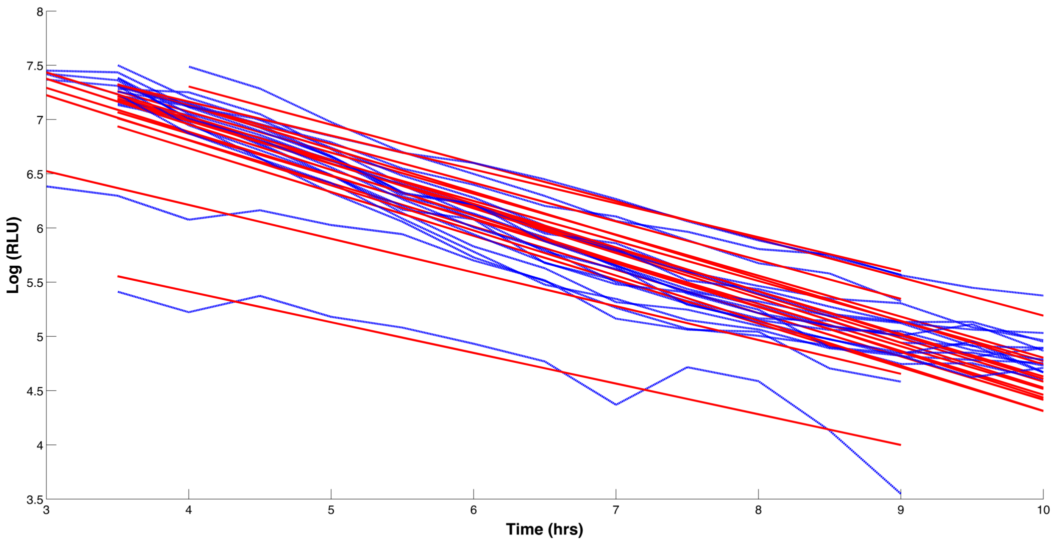

Figure O: log-linear fits to individual data series

2.6 Supplementary Methods: Gibbs Sampler for noise variance estimation

In the MCMC estimation of Fre and LuxEC model parameters, a Gibbs step is introduced for the sampling of noise precision τ, as described below. For LuxAB model, we estimate the noise variance from the replicates data.

Since we are using conjugate (with respect to the form of likelihood) prior for τ, hence, given the data and other parameters, the posterior distribution for τ has a form which can be readily sampled from, see equation 14.

$$\left.\begin{aligned} p(\tau|\theta, Y) \propto \prod_{i=1}^{n} \frac{\tau^(1/2)}{\sqrt{2\pi}} \exp(\frac{-1\tau}{2}(Y_i - \hat(Y)_i)^2) * \tau^(\alpha - 1) exp(-\beta\tau) \\ p(\tau|\theta, Y) \propto \frac{\tau^(n/2)}{\sqrt{2\pi}} \exp(\frac{-\tau}{2}\sum_{i=1}^{n}(Y_i - \hat(Y)_i)^2) * \tau^(\alpha - 1) exp(-\beta\tau) \\ p(\tau|\theta, Y) \propto \tau^(\alpha + \frac{n}{2} - 1) \exp(-\tau(\beta + \frac{\sum_{i=1}^{n}(Y_i - \hat(Y)_i)^2)}{2}) \end{aligned}\right\} \tag{14}$$

As shown in equation 14, posterior distribution for τ is a gamma distribution $\Gamma(\alpha_N, \beta_N)$ with $\alpha_N = \alpha + \frac{n}{2}$ and $\beta_N = \beta + \frac{1}{2}\sum_{i=1}^{n}(Y_i - \hat(Y)_i)^2$. Based on some experimentation with different values, we used $\alpha = 1.75$, and $\beta = 1/1000$ for Fre reaction and $\alpha = 1$, and $\beta = 1/10$ for LuxEC reaction.
